# Draft genome sequence of *Bacillus thuringiensis* C18, a multifunctional isolate from oil palm phyllosphere

**DOI:** 10.1128/mra.01020-25

**Published:** 2025-10-13

**Authors:** Wai Keat Toh, Xin Yen Tor, Pek Chin Loh, Hann Ling Wong

**Affiliations:** 1 Department of Biological Science, Faculty of Science, Universiti Tunku Abdul Rahman 65285, Kampar, Perak, Malaysia; Rochester Institute of Technology, Rochester, New York, USA

**Keywords:** *Bacillus thuringiensis*, biopesticide, draft genome, plant growth promoting, whole genome sequencing

## Abstract

*Bacillus thuringiensis* strain C18, isolated from the oil palm phyllosphere, produces parasporal crystals and exhibits plant growth-promoting (PGP) traits. Its 6.1 Mb draft genome encodes an insecticidal *Spp1Aa1* gene, antimicrobial biosynthetic clusters, and a repertoire of PGP genes, highlighting its dual roles in biocontrol and plant growth promotion.

## ANNOUNCEMENT

*Bacillus thuringiensis* (Bt), a Gram-positive spore-forming bacterium within the *Bacillus cereus sensu lato* group, is well known for insecticidal parasporal crystals that contain Cry and Cyt proteins (1–3). In addition to pest control, some Bt strains promote plant growth through phytohormone production, nutrient solubilization, nitrogen fixation, and siderophore secretion (4, 5).

Here, we present the draft genome sequence of *B. thuringiensis* strain C18 isolated from the phyllosphere of oil palm (*Elaeis guineensis*) leaves grown in soil treated with a plant growth-promoting (PGP) rhizobacteria-based biofertilizer. Strain C18 forms cream to off-white colonies on Nutrient Agar (MilliporeSigma, Burlington, MA, USA), produces parasporal crystals, and exhibits PGP traits, including nitrogen fixation, indole-3-acetic acid (IAA) production, ammonia release, and siderophore secretion, as confirmed by standard PGP assays.

Genomic DNA of strain C18 was extracted from a 5 mL Nutrient Broth (MilliporeSigma) culture grown overnight at 30°C with agitation (220 rpm) using the NucleoSpin Microbial DNA Mini Kit (Macherey-Nagel, Düren, Germany). A total of 100 ng genomic DNA as measured using Qubit dsDNA High Sensitivity Assay Kit (Thermo Fisher Scientific, Waltham, MA, USA) was fragmented to 350 bp size using Bioruptor. The fragmented genomic DNA was used as the input for NEBUltra II Library Preparation Kit (New England Biolabs, Ipswich, MA, USA) and prepared according to the manufacturer’s instructions. Whole-genome sequencing was performed on an Illumina NovaSeq 6000 platform (2 × 150 bp paired-end). A total of 5,404,227 reads were processed with SeqKit v2.3.0 (6), trimmed with Trimmomatic v0.39 (7), error-corrected with Lighter v1.1.3 (8), and assembled with SPAdes v4.0 (9). Genome completeness was assessed with BUSCO v5.4.3 (10) using the *bacteria_odb10* data set, which revealed 99.2% completeness. Default parameters were used for all software, unless otherwise specified.

The draft genome comprised 247 contigs, totaling 6,104,134 bp, with a GC content of 35.3% and an N50 of 57.0 kb at 125× coverage. Annotation with Bakta v1.9.3 (11), eggNOG-mapper (12), and KofamKOALA (13) predicted 6,381 genes, including 6,255 protein-coding sequences (37 pseudogenes and 337 hypothetical proteins), along with 52 tRNAs, 4 rRNAs, 18 ncRNAs, and 1 tmRNA, yielding an overall coding density of 83.5%. CRISPR arrays were not detected.

**Fig 1:**
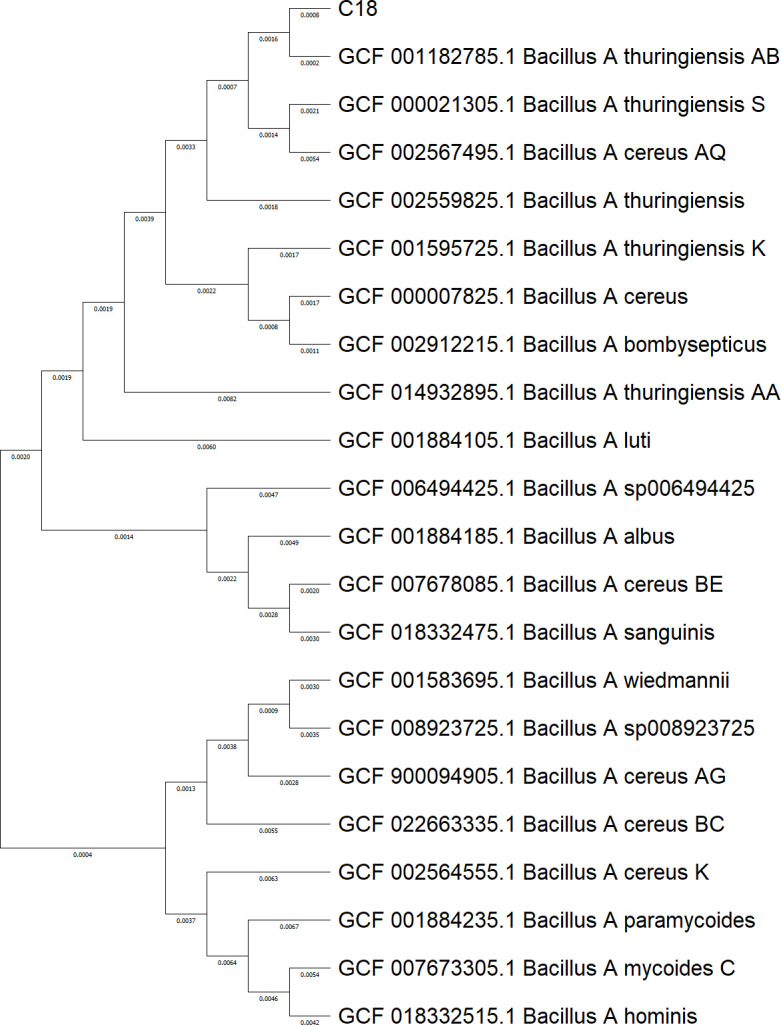
Midpoint-rooted maximum likelihood phylogenetic tree showing the relationship of *B. thuringiensis* strain C18 with closely related species based on the GTDB R220 database. The tree was constructed using concatenated alignment of 75 conserved bacterial proteins. Branch lengths represent the number of substitutions per site and node labels indicate SH-like support values.

Average nucleotide identity (ANI) analysis with FastANI v1.33 (14) against the GTDB R220 database identified *Bacillus_A thuringiensis_AB* (GCF_001182785.1) as the closest relative, and phylogenomic reconstruction with PhyloPhlAn 3 (15) confirmed the placement of strain C18 within the *B. thuringiensis* lineage (Fig. 1).

A Cry-like gene (*Spp1Aa1*) with 80.5% identity to characterized Cry proteins was identified in the genome of strain C18 using BtToxin_Digger v1.0.10 (16) and CryProcessor v1.0 (17), with predicted insect specificity referenced from the Bacterial Pesticidal Protein Resource Center (BPPRC) (https://www.bpprc-db.org/). In addition, secondary metabolite biosynthetic gene clusters predicted by antiSMASH v8.0 (18) included fengycin, bacilysin, bacillibactin, and petrobactin. PGP genes related to nitrogen fixation, mineral solubilization, siderophore production, phytohormone modulation, and stress tolerance were also identified using PGPg_finder v1.1.0 (19).

Collectively, these findings demonstrate the multifunctionality of *B. thuringiensis* strain C18 as both a biocontrol agent and a plant growth promoter, underscoring its potential for sustainable agriculture.

## Data Availability

The project data are available under BioProject under accession number PRJNA1297396, BioSample under accession number SAMN50224141, and Sequence Read Archive under accession number SRR34736292. This Whole Genome Shotgun project has been deposited at DDBJ/ENA/GenBank under the accession JBQPOJ000000000. The version described in this paper is version JBQPOJ010000000.
